# Oily Wastewater Treatment Using Polyamide Thin Film Composite Membrane Technology

**DOI:** 10.3390/membranes10050084

**Published:** 2020-04-28

**Authors:** Sarah Elhady, Mohamed Bassyouni, Ramadan A. Mansour, Medhat H. Elzahar, Shereen Abdel-Hamid, Yasser Elhenawy, Mamdou Y. Saleh

**Affiliations:** 1 Public Works Department of Sanitary and Environmental Engineering, the High Institute of Engineering and Technology in New Damietta, New Damietta 34518, Egypt; 2 Department of Chemical Engineering, Faculty of Engineering, Port Said University, Port Said 42526, Egypt; 3 Materials Science Program, University of Science and Technology, Zewail City of Science and Technology, October Gardens, 6th of October, Giza 12578, Egypt; 4 Chemical Engineering Department, Higher Institute of Engineering and Technology, New Damietta, Damietta 34518, Egypt; 5 Sanitary and Environmental Engineering, Faculty of Engineering, Port Said 42526, Egypt; 6 Department of Civil Engineering, Giza Engineering Institute, Elmoneeb, Giza 12511, Egypt; 7 Department of Chemical Engineering, Egyptian Academy for Engineering and Advanced Technology, Affiliated to Ministry of Military Production, Al Salam city 3056, Egypt; 8 Department of Mechanical Engineering, Faculty of Engineering, Port Said University, Port Fouad 42526, Egypt; 9 High Institute of Engineering and Technology, El-Manzala, Ad Daqahliyah 35642, Egypt

**Keywords:** edible oil, reverse osmosis, COD, oil droplets size

## Abstract

In this study, polyamide (PA) thin film composite (TFC) reverse osmosis (RO) membrane filtration was used in edible oil wastewater emulsion treatment. The PA-TFC membrane was characterized using mechanical, thermal, chemical, and physical tests. Surface morphology and cross-sections of TFCs were characterized using SEM. The effects of edible oil concentrations, average droplets size, and contact angle on separation efficiency and flux were studied in detail. Purification performance was enhanced using activated carbon as a pre-treatment unit. The performance of the RO unit was assessed by chemical oxygen demand (COD) removal and permeate flux. Oil concentration in wastewater varied between 3000 mg/L and 6000 mg/L. Oily wastewater showed a higher contact angle (62.9°) than de-ionized water (33°). Experimental results showed that the presence of activated carbon increases the permeation COD removal from 94% to 99%. The RO membrane filtration coupled with an activated carbon unit of oily wastewater is a convenient hybrid technique for removal of high-concentration edible oil wastewater emulsion up to 99%. Using activated carbon as an adsorption pre-treatment unit improved the permeate flux from 34 L/m^2^hr to 75 L/m^2^hr.

## 1. Introduction

The increased need for edible oil all over the world has resulted in the development of many edible oil plants, leading to the disposal of huge amounts of wastewater. Palm, olive, soybean, cottonseed, and sunflower are considered the main sources for extracting edible oil [1]. Persistent market research (PMR) has reported that the global oil market value is predicted to increase to 130.3 billion US$ by 2024, recording a compound annual growth rate of 5.1%. In 2018, global consumption of vegetable oils was 197.33 million metric tons (MMTs) with an annual production of 203.83 MMTs [2]. Treatment of edible oil industry effluents is considered a major environmental issue. Generally, washing, degumming, de-acidification, deodorization, and neutralization operations are the main sources of oily wastewater discharges. Oily wastewater includes mainly nutrient contents, organic compounds in terms of biological oxygen demand (BOD) and chemical oxygen demand (COD), total suspended solids (TSS), and lipids. Thus, disposal of untreated oily wastewater effluents can result in pollution of soil, water, and air. The waste streams which are emitted without any treatment cause environmental problems such as danger to aquatic life and irreversible damage due to the fact of their high organic concentration and rapid de-oxygenation of water [3,4]. Moreover, wastewater from vegetable edible oil industries contains a higher fraction of slowly biodegradable contents, hence, causing noteworthy environmental issues such as ground and surface water pollution. Accordingly, odor nuisance, water color, and low soil quality can be addressed and reported, taking into consideration environmental issues and human health. Therefore, it is necessary to treat oily wastewater effluents to meet environmental regulations and standards. 

Physical and chemical oily wastewater characteristics depend mainly on the type of edible oil industry where high organic and inorganic pollutants are varied. Numerous methods have been applied for treating pollution in edible oil wastewater [5,6,7,8,9]. Traditional techniques, such as coagulation/flocculation, adsorption, dissolved air flotation (DAF), are normally insufficiently effective to solve the problem, particularly when the oil particles are finely dispersed [10,11,12,13]. Recently, membrane separation and filtration of oily wastewater have gained significant attention because of its ability to remove most chemicals and microbic contents [14]. It is worth mentioning that the quality of the treated water is mostly consistent with the influent variations. Additionally, it can be used in recycling of selected waste streams for different applications. Membrane technology is used to obtain stable permeate and rejected stream quality which can be reused and recycled in desired applications [15,16,17,18,19,20]. Microfiltration and ultrafiltration processes to treat oily wastewater have previously been reported [21,22]. Masoudnia et al. (2015) employed microfiltration (MF) using polyvinylidene fluoride (PVDF) membrane for wastewater from the edible oil industry [23]. However, it was found that membrane performance decreased with time due to the fact of fouling issues [24]. Membrane fouling may include pore blocking, chemical degradation, and cake formation on the surface of membrane due to the presence of microbes or impurities gathering in the form of inorganic and organic matters. Additionally, this process leads to shorter life expectancy of the membrane. Polymeric and ceramic membranes have been employed in oily wastewater treatment [25,26]. Most organic membranes offer several benefits such as low cost and high removal efficiency. However, they cannot withstand the high temperature, pressure, and fouling that limit the membrane regeneration [27]. Membrane fouling as a result of the accumulation of impurities has always been a challenge and limits membrane applications [28]. Chemicals to clean fouling increase the cost and energy demand. Coupling of feed water pretreatment to the membrane unit is a possible solution to reduce the fouling issue [29]. Thus, in order to decrease membrane fouling, pre-treatment of the unit is often preferred (including coagulation, adsorption, filtration, or oxidation) [30,31,32]. It is supposed that oil droplets and colloids are destabilized and gathered, forming larger flocs within coagulation pretreatment units which can be removed easily by membrane filtration [33]. 

Modification of the membrane structure to be more hydrophilic is another solution to mitigate fouling formation [14]. Various membrane treatment types (namely, UF, Nanofiltration (NF) anaerobic membrane system, RO, and MF) have been reported as oily wastewater treatments [34]. The COD and turbidity measurements of permeate stream must be measured in order to estimate the optimal conditions for oily wastewater treatment [35]. Šereš et al. (2016) have successfully removed up to 99% of turbidity and 85% of COD from oily wastewater using microfiltration ceramic membrane. Its pore size (200 nm) and transmembrane pressure operated up to 3 bar [36]. Reverse osmosis is able to treat oily wastewater. It is generally used to avoid membrane fouling problems [34]. Stoller et al. (2016) used hybridizing membrane systems, including UF, NF, and RO, for the treatment of olive oil processing wastewater [37]. Azmi et al. studied a UF and RO hybrid membrane system on palm oil/water emulsion. Removal of BOD and turbidity up to 98.9% and 99%, respectively, were obtained [38]. In another study, the same research team used sand filtration and coagulation/flocculation before the membrane unit for oily wastewater treatment [39]. A remarkable reduction in COD (95%) and BOD (95%) was recorded. In this study, an activated carbon adsorption unit was used for pretreatment. The present investigation aimed to identify the optimum conditions allowed for the permeate stream suitable for recycling or to be discharged into water sewage with no risk of toxicity to the ecosystem. Coupling of the adsorption unit to RO was investigated in a hybrid mode. The treatment processes were compared based on their efficiency in COD and turbidity removals. It was concluded that this coupling of treatment units was able to purify edible oil wastewater emulsion and treated water to meet standards. 

## 2. Materials and Methods 

Polyoxyethylene sorbatin monolaurate “Tween 20” (C_58_H_11_O_26_, MWt = 1227.72 g/mol) was used as a surfactant. Oily wastewater was synthesized by mixing 5 mLof soybean oil and 1 L of distilled water in the presence 1 mL of Tween 20. The oily wastewater emulsion was mixed mechanically for 3.5 h at a mixing rate of 300 rpm. The stability of the oily wastewater emulsion was observed after 24 h. The mixture showed a uniform and stable white emulsion. The PA-TFC RO membrane in a spiral wound structure was investigated. The Membrane model (TW 30-1812-75) was installed—length: 0.26; diameter: 0.05 m; maximum service temperature and pressure: 45 °C and 1.034 MPa, respectively; applied pressure: 0.86 MPa; and allowable range of pH: 2 to 10. The pores’ size ranged from 0.67 nm to 0.78 nm [40]. The maximum flow rate of was 0.0076 m^3^/min. The granular activated carbon (GAC) filter was installed as an adsorption pretreatment unit. The column dimensions were 63.5 mm and (height) 254 mm. The GAC filter was fixed to reduce total suspended solids and chemicals resulting in undesired tastes and odors and volatile organic chemicals (VOCs). The GAC is capable of delivering up to 0.0038 m^3^ per minute of treated water. A high-pressure pump (PKM60) with dimensions 0.0254 m × 0.0254 m, Q_max_ “0.04 m^3^/min”, and “2850 rpm” was installed to pump the water–oil emulsion across the membrane surface. An analysis of oil wastewater and treated water was conducted in terms of turbidity, oil and grease, and COD.

### 2.1. Measuring and Analysis

#### 2.1.1. Chemical Oxygen Demand (COD)

Chemical oxygen demand for all oily wastewater samples (feed, permeate, and rejection) was measured by COD photometer (LaMotte, Chestertown, MD, USA). Sample (0.2 mL) was added into digested solution (High range: 200–15,000 mg/L) consisting of a strong oxidizing agent (potassium dichromate), mercuric sulfate, and sulfuric acid. The water mixture was then heated for 120 min in a COD heater at 150 °C. By the end of oxidation, the COD was measured using a spectrophotometer.

#### 2.1.2. Turbidity

The samples of feed, permeate, and rejection were analyzed for total suspended solids in terms of turbidity using a turbidity meter (Lovibond meter, Lovibond company, Sarasota, FL, USA) expressed as nephelometric turbidity units (NTUs).

#### 2.1.3. Thermogravimetric Analysis (TGA)

Thermal stability of polyester support, polysulfone, and polyamide layers were characterized using TGA (TGA Q500 V20.10 Build 36, TA Company, Tokyo, Japan). Thermogravimetric analysis is considered a proper method to quantitatively examine the thermal stability of polymeric membrane materials.

#### 2.1.4. Fourier-Transform Infrared Spectroscopy (FTIR)

The membrane model (TW 30-1812-75) was investigated using FTIR analysis (Avatar 370 Nicolet Spectrometer, Madison, CA, USA) to characterize and identify chemical bonds.

#### 2.1.5. Scanning Electron Microscopy (SEM)

The thin film composite RO membrane was freeze-fractured and gold coated using an S150A SPUTTER COATER SEM (Edwards High Vacuum = 7.5 mbar). Surface morphology and cross-sections of TFC membranes were investigated using scanning electron microscopy (SEM, Quanta 250 FEG, FEI Company, Tokyo, Japan).

#### 2.1.6. Mechanical Testing 

The strength of the RO membrane was measured using a (Tinius Olsen H25KS, Shanghai, China) tensile instrument by providing load coaxial style.

#### 2.1.7. Average oil Particle Size

The size distribution of oil droplets in the oily wastewater feed was determined using a laser particle size analyzer (Nicomp Nano size, 380 ZLS, Santabarbara, CA, USA).

#### 2.1.8. Dynamic Wetting 

Each sample was cut to a size of 10 mm × 50 mm with sharp scissors. The TFC-PA specimen was immersed into oily wastewater and water samples. The weight of the absorbed water was measured. The dynamic water absorption was plotted versus time.

#### 2.1.9. Contact Angle

To study the effect of edible oil on the hydrophobicity of the PA-THF RO membrane, the surface contact angle of the membrane was measured based on the sessile drop method via a contact angle measuring instrument (OCA 15EC contact angle model, GmbH, Filderstadt, Germany). The reported results were the average contact angle of deionized water droplets and oily wastewater at five different locations on each sample.

#### 2.1.10. Oil and Grease

The oil concentration was determined using the hexane extraction gravimetric method. One hundred and twenty-five milliliters of oily wastewater samples were added to ethanol (20 mL) and sulfuric acid (20 mL). Mixtures were well shaken in a separation funnel. After fifteen minutes, two layers were separated, and the supernatant layer containing the oil (organic layer) was collected. The weight (wt) of the beaker before and after separation was recorded. Then, the oil concentration was calculated as given in Equation (1).

(1) $$\mathrm{Oil}\;\mathrm{concentration}\;=\frac{\mathrm{wt}\;\left(\mathrm{mg}\right)\;}{\mathrm{volume}\;\mathrm{of}\;\mathrm{sample}\;\left(\mathrm{ml}\right)\times 1000}$$

### 2.2. Experimental Setup and Operation

**Figure 1:**
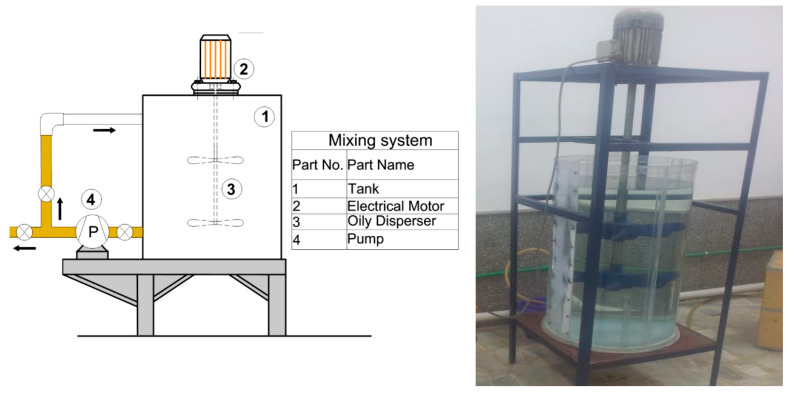
Mixing tank for preparation of nano-emulsion oil in water.

The tank, made from acrylic for mixing the water–oil emulsion, is shown in Figure 1. The tank’s dimensions were 0.4 m in diameter and 0.7 m in height with a total volume of 0.05 m^3^ and a rotational speed of 300 rpm for the stainless-steel mixer.

**Figure 2:**
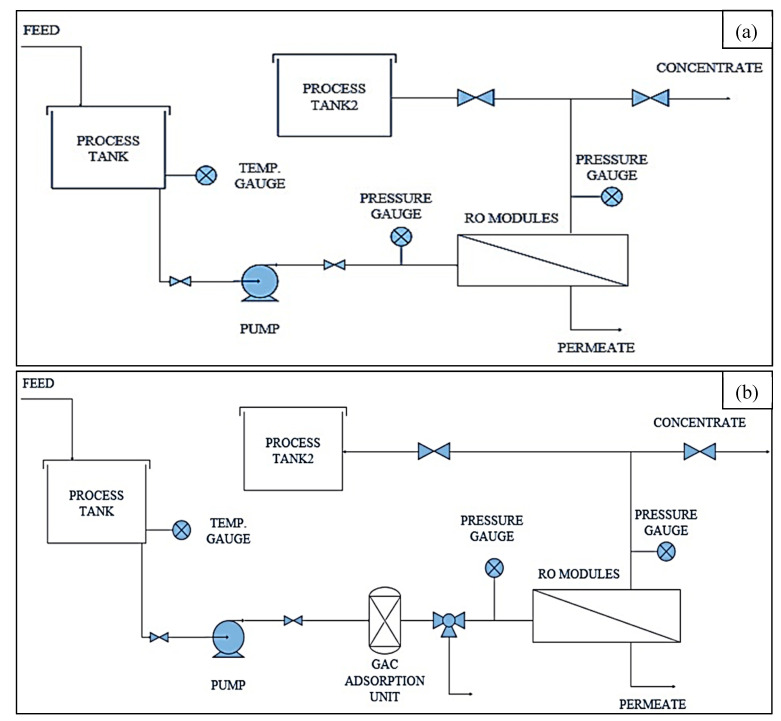
The diagram of an edible oil wastewater treatment unit using (**a**) a polyamide (PA) thin film composite (TFC) reverse osmosis (RO) membrane (**b**) pretreatment unit with a powder carbon filter.

Figure 2a shows the schematic diagram of the PA-TFC RO membrane filtration unit and the facilities. The membrane was washed using de-ionized water for fifteen minutes before the treatment process to ensure that the RO had reached maximum force. The synthetic wastewater/oil emulsion was fed in mechanically stirred tanks, 0.04 m^3^, in the presence of surfactant (Tween 20). The oily wastewater was pumped to the RO unit under a constant pressure of 8.5 bar. The oil concentration in wastewater varied from 3 g/L to 6 g/L. The treated water was collected from the RO unit, determined by collected permeate water in vessel. The permeate flow rate was measured regularly to calculate flux. The permeate, rejected, and feed water contents were tested for COD, oil and grease, and turbidity. The rejected wastewater stream was analyzed to determine oil content in the oily wastewater. To control the membrane fouling, a pre-treatment adsorption unit of activated carbon was installed. In the feed pretreatment unit, adsorption GAC was applied to remove suspended solids and organic constituents. The pretreatment process plays a crucial role for treatment process of high concentration edible oil wastewater in oil industries that would block the membrane and reduce its lifetime. A process flow diagram (PFD) of the pre-adsorption unit and membrane filtration process are shown in Figure 2b. After oily wastewater treatment using the hybrid system (i.e., GAC and RO membrane), the treated water sample was tested for possible irrigation or discharge into the sewage network. The oil contents were determined in the rejected stream for recycling highly concentrated oily wastewater streams in the production of soap as a step towards a zero discharge system [41]. Turbidity and COD were measured in each process to study the efficiency of oil removal.

#### Percentage Oil Rejection and Flux

In the RO membrane unit, the separation efficiency was determined in terms of rejection (%) of COD, turbidity or oil and grease as given in Equation (2) [5]:

(2) $$R\left(\%\right)=\frac{\mathrm{CF}-\mathrm{CP}}{\mathrm{CF}}\times 100$$

The CP is the concentration of oil in permeate stream; CF is the oil concentration in the feed.

The water flux (Jω) is the volume of permeate (V) that is collected per unit membrane surface area (a) per unit time (t). Water flux is determined as given in Equation (3):

(3) $$J\omega =\frac{V}{a\times t}$$

## 3. Results and Discussion

### 3.1. Membrane Characterization

#### 3.1.1. Surface Morphology

**Figure 3:**
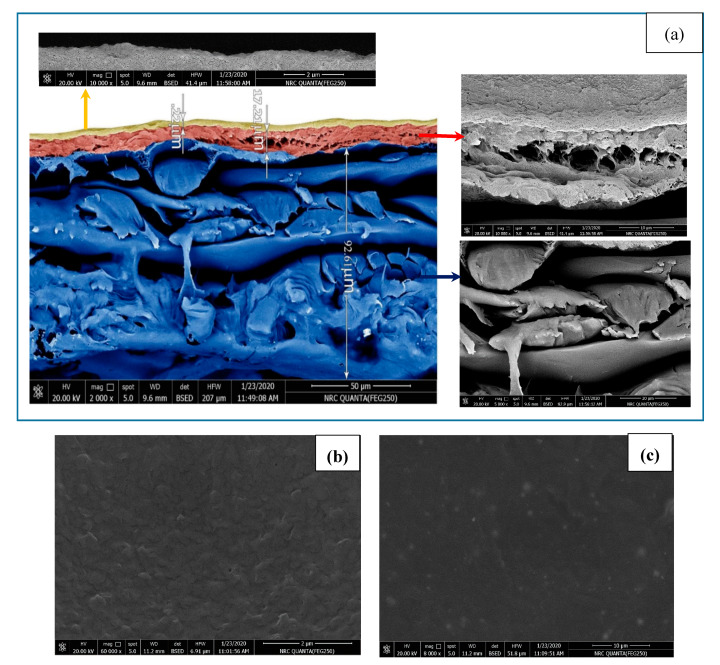
(**a**) Cross-section of an RO membrane, (**b**) surface morphology before oily wastewater treatment, and (**c**) surface morphology after oily wastewater treatment.

Polyamide thin film composite contains three layers of thin film PA, polysulfone, and a supported layer of polyester. The SEM images of the TFC membrane were investigated before and after oily wastewater treatment to analyze microscopic structure of the membrane. Cross-sectional images of the TFC were taken after the cutting with freeze-fracturing. Figure 3a shows cross-sectional images which show the RO polyamide membrane consisting of three layers: a thin selective layer on the upper surface (0.22 µm), a microporous interlayer (about 17.21 µm), and polyester acting as structural support (92.61 µm). The SEM micrographs of pristine membrane are shown in Figure 3b. Membrane surface morphology after wastewater treatment is shown in Figure 3c. It was found that oil droplets absorbed on the surface of the TFC membrane. In the absence of the GAC unit, most of the membrane pores were blocked, resulting in a decrease in membrane flux. Zhao et al. (2015) reported similar results on polyvinylchloride ultrafiltration membranes blocking in oily wastewater treatment [42].

#### 3.1.2. Contact Angle

**Figure 4:**
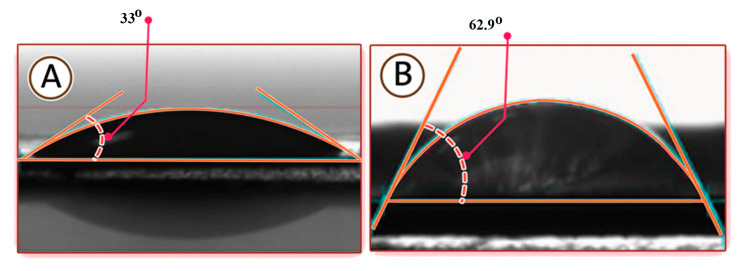
Contact angles of (**A**) water and (**B**) oil on the membrane surface.

Hydrophilicity is one of the most crucial parameters that influences the permeability and antifouling process of the membrane surface. A low contact angle refers to the high hydrophilicity of the surface. Contact angles of the TFC membrane were measured using advancing contact angle methods. As shown in Figure 4, the contact angle of water on the surface was 33°, while the average contact angle of oily wastewater on the membrane surface was 62.9°. These results suggest that the membrane in water was more hydrophilic than oil. 

#### 3.1.3. Mechanical Test

**Figure 5:**
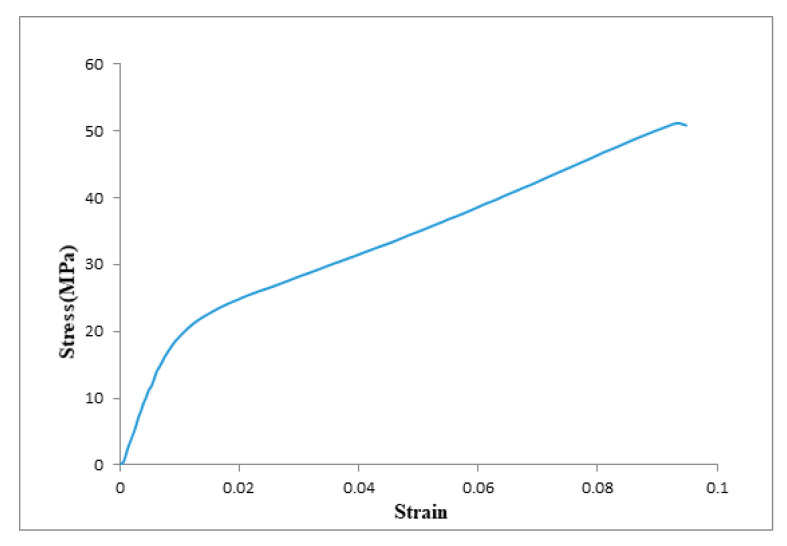
The stress–strain curve for PA-TFC reverse osmosis membrane.

Figure 5 shows the results of the stress–strain behavior of the TFC membrane. The tensile strength was found to be 52 MPa, and the Young’s modulus was 1000 MPa. The mechanical testing results are listed in Table 1. 

#### 3.1.4. Thermogravimetric Analysis (TGA)

**Figure 6:**
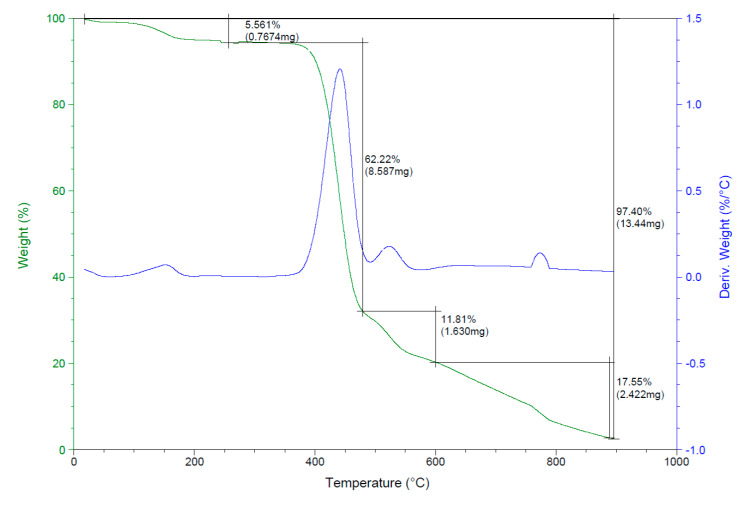
TGA for the reverse osmosis membrane.

Thermogravimetric analysis (TGA) of the reverse osmosis membrane was performed under nitrogen atmosphere as shown in Figure 6. Four weight-loss stages can be observed in the TGA curve. Weight loss at an average temperature 100 °C can be attributed to water loss. The second stage at an average temperature 304–551 °C refers to the thermal degradation range of the supported polyester layer. The third stage at an average temperature 450–550 °C can be attributed to polyamide loss. The fourth stage shows the weight loss of the polysulfone layer, starting from 590 °C. 

#### 3.1.5. Fourier Transform-Infrared Spectroscopy FTIR

**Figure 7:**
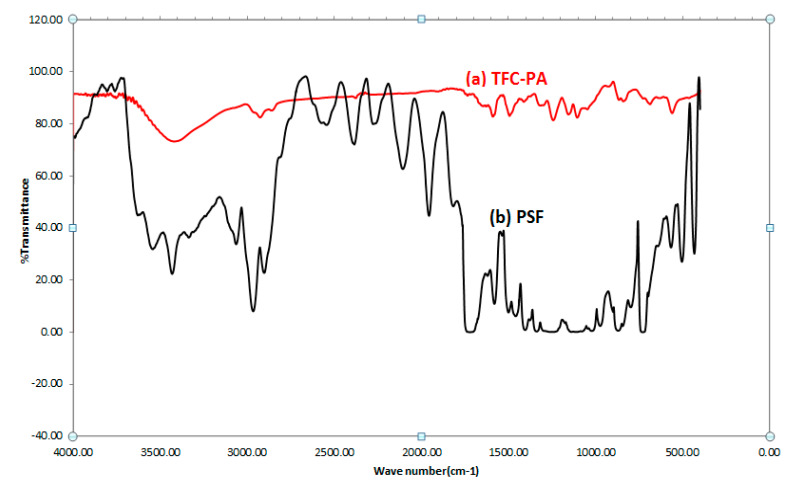
FTIR spectra for TFC-PA membrane and polysulfone (PSF) support layer.

The FTIR was used to analyze the chemical composition of the top surface of membrane (PA), and PSF and polyester-supported layer. The infrared spectra of TFC membrane showed the main characteristic peaks of PA layer and polysulfone-supporting layer as shown in Figure 7 and listed in Table 2.

### 3.2. Effect of Oil Particle Size on Separation Efficiency

**Figure 8:**
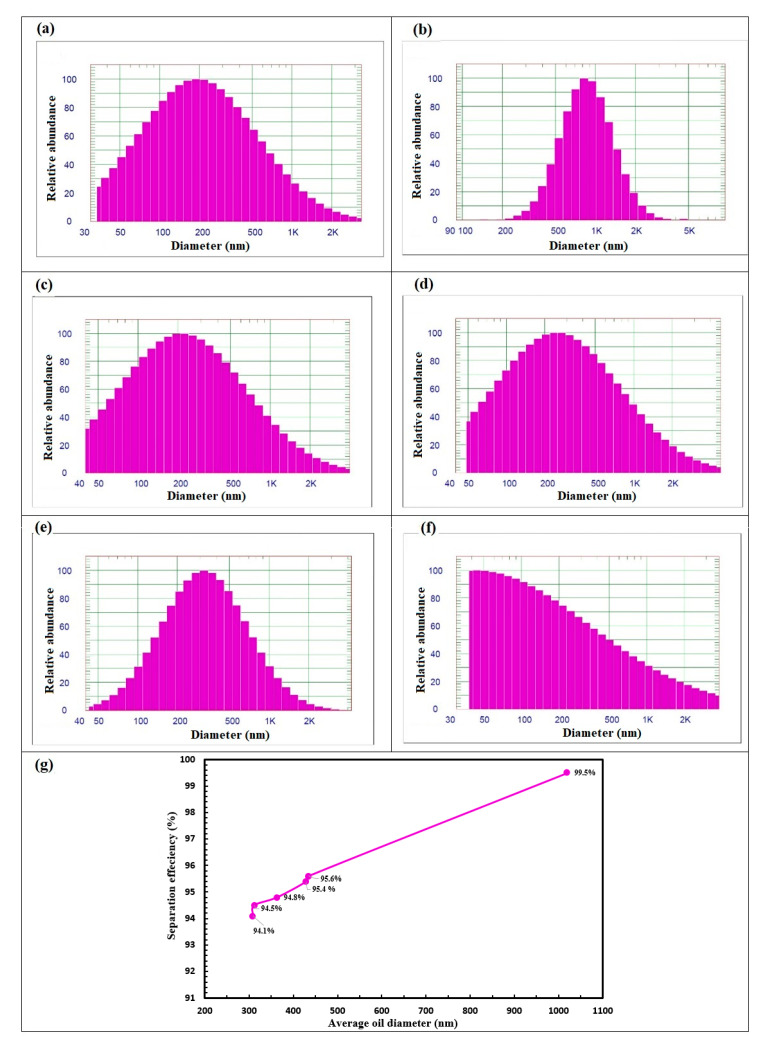
Effect of oil particle size on separation efficiency, average oil droplet size (**a**–**f**) and (**g**) effect of average oil droplet size on membrane separation.

Oil particles that are captured on the PA-TFC RO membrane surface affect separation efficiency across membrane filtration. The PA-TFC RO membrane’s capacity may vary depending on the particle size variation in the oily wastewater as shown in Figure 8. The distribution of oil droplets’ size was measured for six samples of emulsion to study the effect of oil particle size on the separation efficiency. It was found that the separation performance improves as the oil particles’ sizes increase. This can be attributed to the coalescence of oil particle droplets where these are retained on thin film membrane. Thus, rejection efficiency increased from 94% to 99% with the increase of oil concentration as well as the average droplet size. Similar trends were observed by Chakrabarty et al. [43] and Mittal et al. [44].

### 3.3. Dynamic Wetting

**Figure 9:**
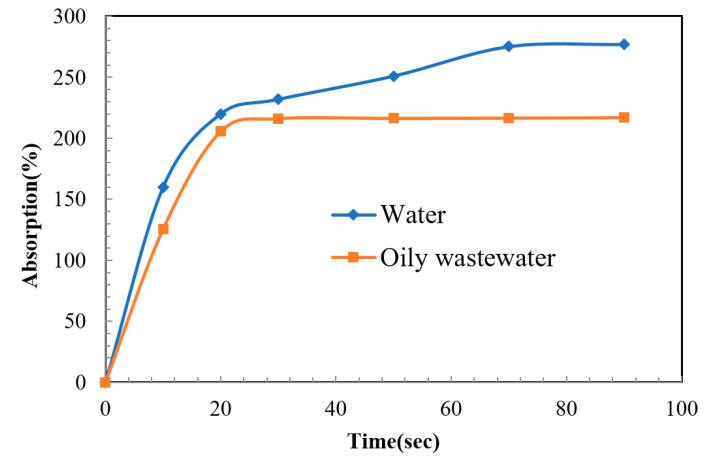
Water and oily wastewater absorption of the thin film composite membrane.

All membranes demonstrated water absorption curves that rose sharply within 20 s. Further immersion occurred after 20 s up to 90 s, inducing a plateau in all curves. Early in the immersion period, instantaneous water absorption was observed for all membranes. After 20 s all curves plateaued, showing that all membranes could only absorb a certain number of water molecules. This observation confirms the hydrophilic behavior of the TFC membrane. The absorption curve of the TFC membrane can be seen in Figure 9. Oil contents considerably decreased the water absorption properties of the RO membrane. This can be attributed to the higher contact angles.

### 3.4. Effect of Time on Permeate Flux

**Figure 10:**
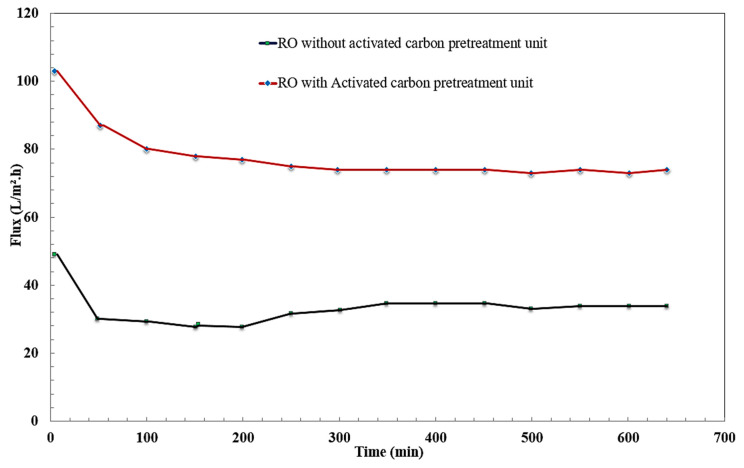
Effect of time separation on flux.

The fluxes of permeate oily wastewater were taken every 50 min, starting from 5 min. Figure 10 shows that the initial flux in case of RO and RO and adsorption pretreatment hybrid system were 34 L/m^2^·h and 75 L/m^2^·h, respectively. The initial flux was the maximum value at constant pressure 8.5 bar. This phenomenon can be attributed to the lowest value of mass transfer resistance at the beginning. At the membrane surface, permeate flow rate reduces with time and concentration polarization increases. A decrease in flux was recorded as a result of increased mass transfer resistance, so fouling is often considered in the form of decreasing permeate flux over time [45,46,47,48,49]. Membrane blocking leads to a decrease in membrane performance because of suspended solids accumulating on the outer layer. This could be observed in the RO unit without an adsorption unit. Differences in fouling behavior between the oily wastewater treatment using RO and the RO hybrid system were expected as a result of the differences in the rate of fouling. 

### 3.5. Effect of Oil Concentration on Separation Efficiency

**Figure 11:**
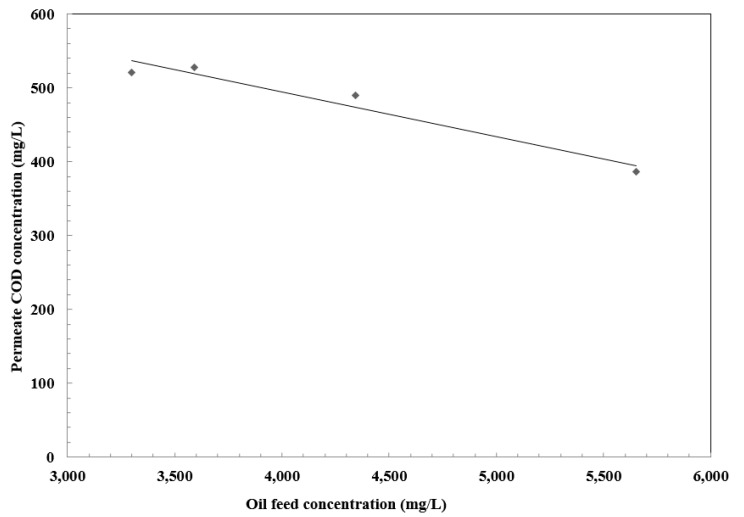
Effect of feed concentration on permeate COD.

**Figure 12:**
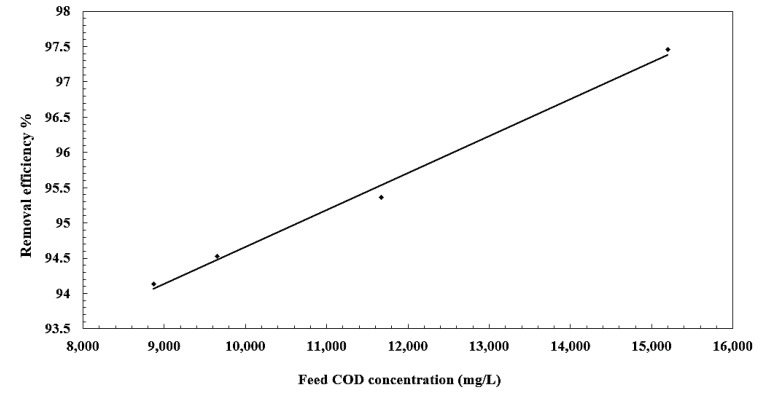
Effect of feed concentration on removal efficiency.

The effect of the oil concentration on the separation performance was determined at a constant pressure of 0.85 MPa, as listed in Table 3, which describes the maximum concentration of suspended solid in terms of turbidity and COD removal. The results are presented in Figure 11 and Figure 12, showing that the oil removal increases with an increase in feed concentration. Separation efficiency improves with the increase in oil concentration which can be attributed to the formation of larger oil droplets. Furthermore, oil droplets separated by the membrane form a gel layer on the membrane surface [43]. Thus, removal can be improved. However, the flux decreases in the absence of an adsorption pretreatment unit. The average removal of oil in terms of COD reached 97.4%. The performance of membrane showed high COD removal. The COD of the permeate stream was within the allowable levels (1100 ppm) for the discharge of wastewater into sewage networks.

### 3.6. Effect of Adsorption Pretreatment unit on Membrane Filtration Performance

Permeate flux decreased as a result of membrane fouling and concentration polarization. Using activated carbon pretreatment unit led to a decrease in suspended solids’ load on membrane filtration. Oil removal improved to 99.9% in the presence of a pre-treatment unit as listed in Table 4. Oil concentration in terms of COD in the permeate stream was less than 100 mg/L which meets the national discharge standards of wastewater.

## 4. Conclusions

The reverse osmosis thin film composite membrane showed good results in the treatment of oily wastewater produced from the edible oil industry. Removal of turbidity, COD, and oil removal at fixed flux was achieved using RO and an adsorption unit hybrid system. An activated carbon unit as a pretreatment unit showed better efficiency and higher permeate flux. Membrane filtration using a reverse osmosis membrane was conducted to remove edible oil and suspended solids from oily wastewater. An RO membrane was characterized using FTIR, SEM, contact angle, mechanical analysis, and TGA. The PA-TFC membrane showed a promising separation process for treatment of wastewater effluent from edible oil. Significant removal of COD and turbidity can be accomplished using RO. Higher flux and better membrane performance can be achieved using an RO membrane filtration coupled with an activated carbon unit. A concentration of oil up to 6000 mg/L can be effectively improved to 99.9%. 

## Figures and Tables

**Table 1:** Mechanical properties of polyamide membrane.

Property	Polyamide Thin Film Composite
Tensile strength, MPa	51.15
Young’s modulus, MPa	1000

**Table 2:** The main characteristic peaks for PA and PSF.

PA		PSF	
Range (cm^−1^)	Assignment	Range (cm^−1^)	Assignment
840.39	aromatic hydrogen, isolated	727.89	Aromatic hydrogen
1105.05	-	870.12	Hydrogen deformation of para-substituted phenyl groups.
1155.67	C–N bending	1248.53	C–O–C asymmetric stretching vibration of the aryl–O–aryl group
1242.98	C–N bending	1581.03	Aromatic in-plane ring bend stretching vibration
1493.59	Aromatic ring breathing	3064.54	O–H aromatic stretching
1590.13	C=O band of an amide group, C–N stretching, and C–N–C deformation vibration in a secondary amide group	-	-
3420.93	N–H (and O–H)	-	-
3647.56	O–H aromatic stretching bands	-	-

**Table 3:** Effect of oil concentration on the properties of treated water.

Feed COD Concentration (mg/L)	Permeate COD Concentration (mg/L)	Separation Efficiency %	Feed Turbidity (NTUs)	Permeate Turbidity (NTUs)
5653	386	97.4	990	1.28
4343	490	95.35	980	1.22
3591	528	94.53	870	1.84
3299	521	94.12	850	1.6

**Table 4:** Effect of pretreatment on permeate characteristics.

Oil Concentration of Feed (mg/L)	Oil Concentration of Permeate before Pretreatment (mg/L)	Oil Concentration of Permeate after Pretreatment and RO (mg/L)	Rejection %
5653	105	4.8	99.91
4343	219	5.3	99.87
3591	186	4.3	99.88
3299	235	3.2	99.90
